# Supplementary material for: LUBAC prevents lethal dermatitis by inhibiting cell death induced by TNF, TRAIL and CD95L
Source: Nat Commun. 2018 Sep 25;9:3910. doi: 10.1038/s41467-018-06155-8 (PMC6156229; doi:10.1038/s41467-018-06155-8)
Supplement: Supplementary file 1 — Supplementary Information [file 41467_2018_6155_MOESM1_ESM.pdf]

Supplementary Information

LUBAC prevents lethal dermatitis by combined inhibition of cell death induced by TNF, TRAIL and CD95L

Lucia Taraborrelli[1,10], Nieves Peltzer[1,10], Antonella Montinaro[1], Sebastian Kupka[1], Eva Rieser[1], Torsten Hartwig[1], Aida Sarr[1], Maurice Darding[1], Peter Draber[1], Tobias L. Haas[2], Ayse Akarca[3], Teresa Marafioti[3], Manolis Pasparakis[4], John Bertin[5], Peter J. Gough[5], Philippe Bouillet[6,7], Andreas Strasser[6,7], Martin Leverkus[8,9], John Silke[6,7] and Henning Walczak[1,*]

[1]Centre for Cell Death, Cancer, and Inflammation (CCCI), UCL Cancer Institute, University College London, 72 Huntley Street, London WC1E 6DD, UK

[2]Department of Hematology, Oncology and Molecular Medicine, Istituto Superiore di Sanità, Rome, Italy

[3]Department of Cellular Pathology, University College London, 21 University Street, WC1E 6DE, London, UK

[4]Cologne Excellence Cluster on Cellular Stress Responses in Aging-Associated Diseases (CECAD), and Center for Molecular Medicine (CMMC), University of Cologne, Cologne, Germany

[5]Pattern Recognition Receptor Discovery Performance Unit, Immuno-Inflammation Therapeutic Area, GlaxoSmithKline, Collegeville, PA 19422

[6]The Walter and Eliza Hall Institute of Medical Research, 1G Royal Parade, Parkville, Victoria 3052, Australia

[7]The Department of Medical Biology, The University of Melbourne, Parkville, Victoria 3050, Australia

[8]Department of Dermatology & Allergology, University Hospital of RWTH Aachen University, 52074 Aachen, Germany

[9]Deceased

[10]These authors contributed equally.

*Correspondence to: Henning Walczak, PhD; E-mail: h.walczak@ucl.ac.uk; Phone +44 207 679 6471

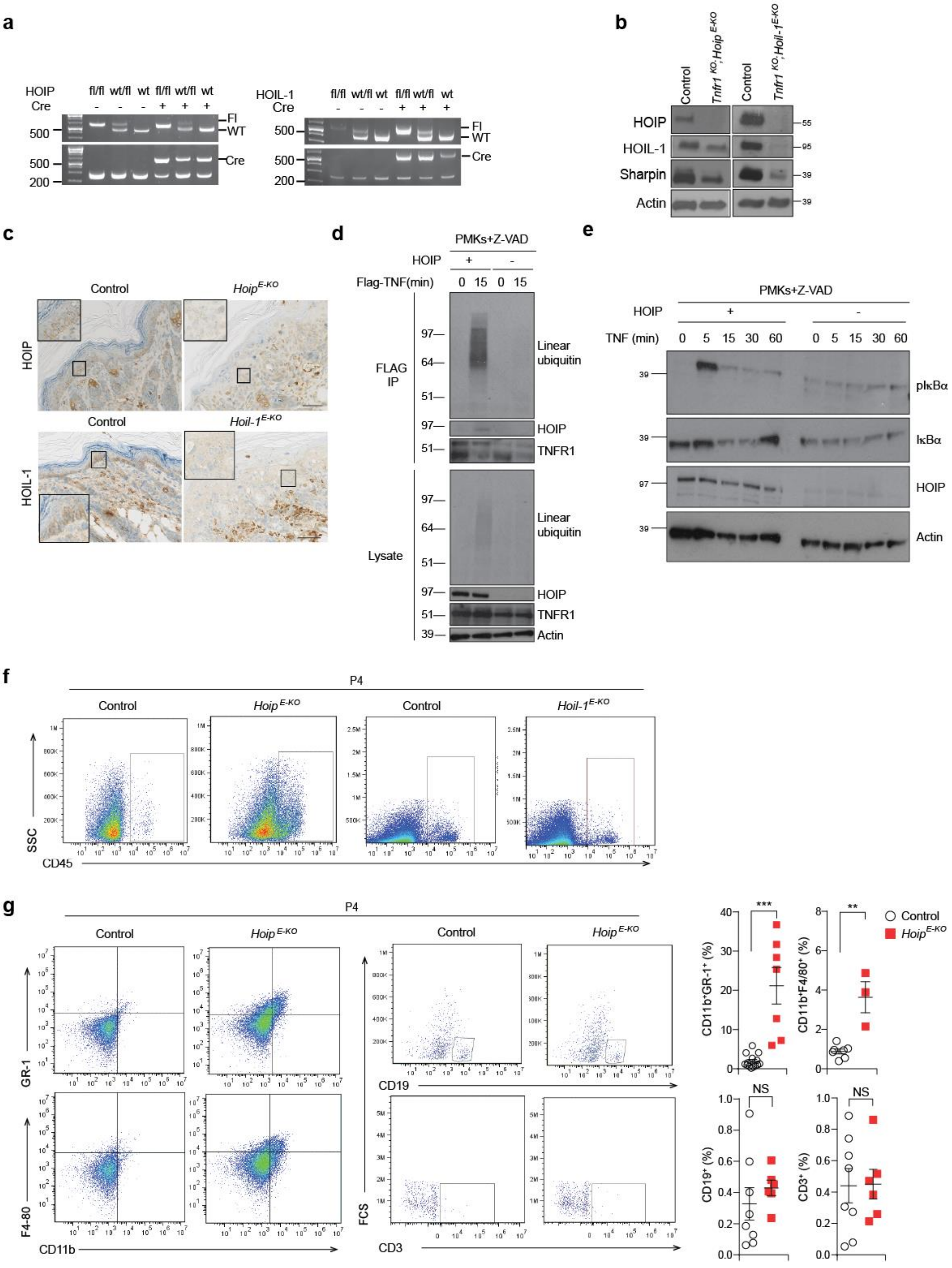

Supplementary Figure 1. Generation and characterisation of mice deficient for HOIP and HOIL-1 in keratinocytes. a, PCR genotyping of DNA isolated from the ear punch of mice with the indicated genotypes. **b,** Western blot analysis of LUBAC components in PMKs derived from mice of the indicated genotypes. Images are representative of two independent experiments. **c,** Representativimages of skin sections stained with antibody against HOIP (upper panel) and HOIL-1 (lower panel) at P4. Scale bar, 50 µm. **d,** Endogenous TNFR1 complex I pull-down was performed by FLAG IP in PMKs derived from control (+) or *Hoip*$^{E\text{-}KO}$ (-) mice cultured in presence of the caspase inhibitor ZVAD-fmk and stimulated

with FLAG-TNF. Lysates and IP were analyzed by Western blotting for the indicated proteins. **e,** Western blot analysis of the indicated proteins in whole-cell lysates from PMKs derived from control (+) and *Hoip*$^{E\text{-}KO}$ (-) mice following stimulation with His-tagged TNF [100 ng/ml] for different time points (min). **f,** Gating strategy of $CD45^+$ cells in skin of the indicated genotypes at P4. Quantification shown in Fig. 1d. **g,** Gating strategy and percentage of the indicated immune cells in skin samples from mice of the indicated genotypes at P4. Data are presented as mean values ± s.e.m (n= at least 3 per genotype). $^{**}P \leq 0.01$, $^{***}P \leq 0.001$, NS: not significant. See sequential gating strategy (f, g) in Supplementary Fig. 11.

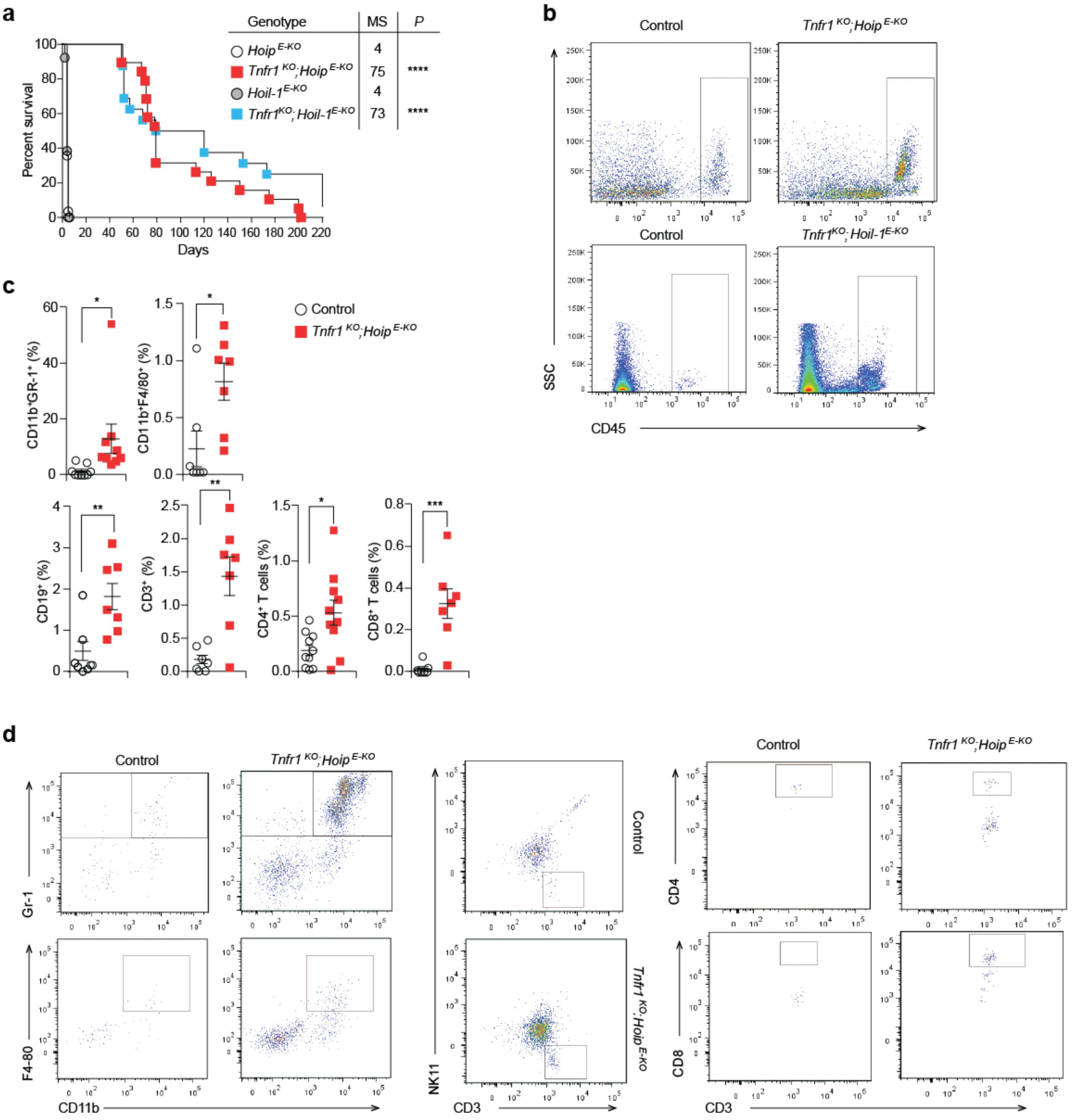

Supplementary Figure 2. TNFR1 deficiency in *Hoip*$^{E-KO}$ and *Hoil-1*$^{E-KO}$ mice results in skin inflammation in adulthood. a, Kaplan-Meier survival curve of mice of the indicated genotypes. Comparisons between *Hoip*$^{E-KO}$ (*n*=10) and *Tnfr1*^{KO};*Hoip*$^{E-KO}$ (*n*=27) and *Hoil-1*$^{E-KO}$ (*n*=12) and *Tnfr1*^{KO};*Hoil-1*$^{E-KO}$ (*n*=20) mice were submitted for statistical analysis. MS: median survival, ****$P \leq 0.0001$. **b,** Gating strategy of CD45^{+} cells in skin of the indicated genotypes at P70. Quantification shown in Fig. 2d. **c, d,** Percentage and gating strategy of the indicated immune cells in skin samples from mice of the indicated genotypes at P70. Data are presented as mean values ± s.e.m (*n*= at least 7 per genotype). *$P \leq 0.05$, **$P \leq 0.01$, ***$P \leq 0.001$. Control mice represent a pool of *Tnfr1*^{KO};*Hoip*$^{fl/fl}$;*K14-Cre-* and *Tnfr1*^{KO};*Hoip*$^{fl/wt}$;*K14-Cre+* mice (b-d) and *Tnfr1*^{KO};*Hoil-1*$^{fl/fl}$;*K14-Cre-* and *Tnfr1*^{KO};*Hoil-1*$^{fl/wt}$;*K14-Cre+* mice (b). See sequential gating strategy (b, d) in Supplementary Fig. 12.

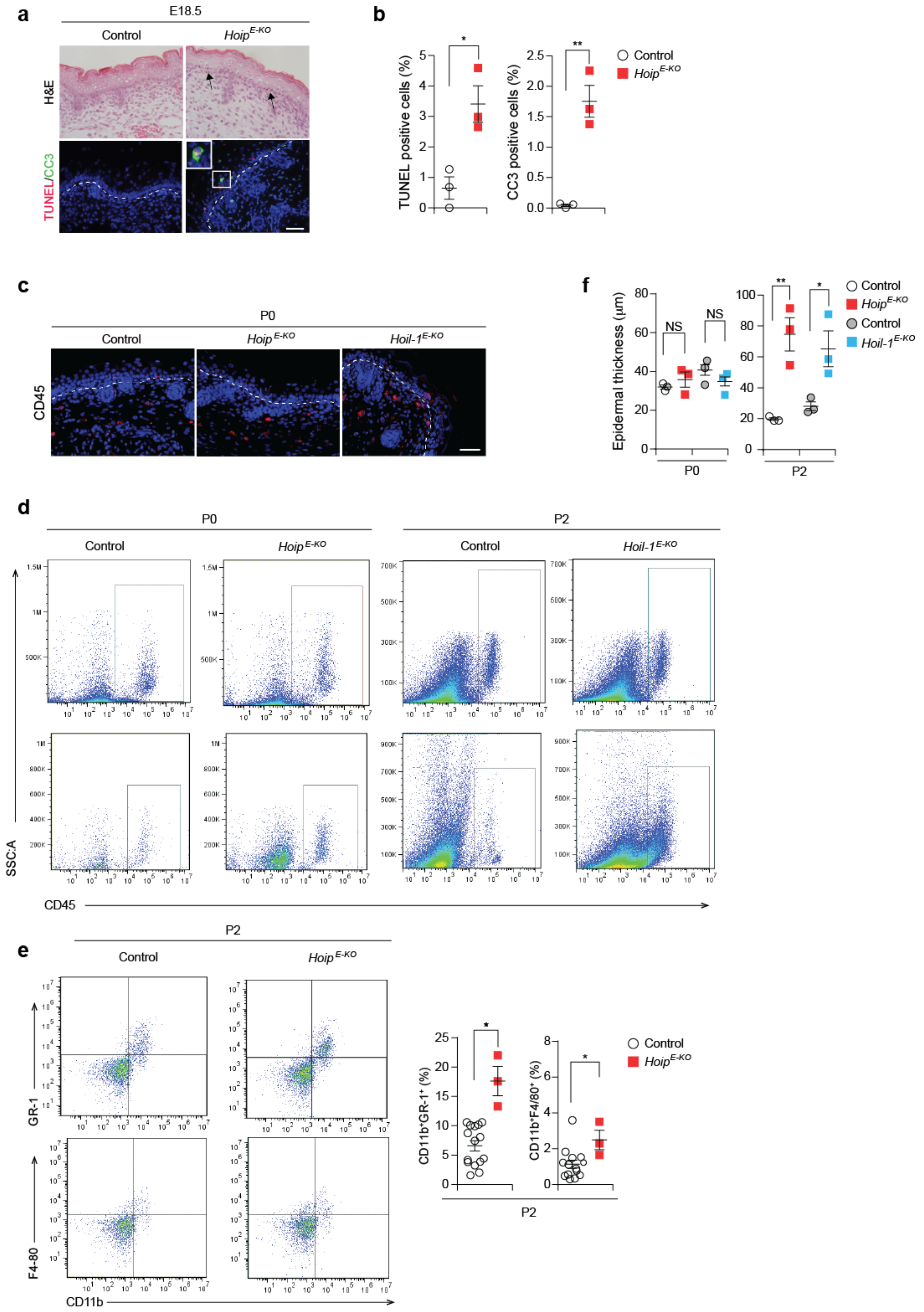

Supplementary Figure 3. Analysis of *Hoip*$^{E\text{-}KO}$ and *Hoil-1*$^{E\text{-}KO}$ mice at different time points. a, b, Representative images of skin sections from *Hoip*$^{E\text{-}KO}$ mice with the indicated stainings (a) and

corresponding quantification (b), TUNEL (red) and cleaved caspase-3 (CC3) (green) at E18.5. Nuclei were stained with DAPI (blue). White dashed lines indicate boundary of epidermis (above) and dermis (below). Arrows indicate pyknotic nuclei. Scale bars, 50 µm. Data are presented as mean values ± s.e.m (*n*=3 mice per genotype). **P*≤ 0.05, ***P*≤ 0.01. **c,** Representative images of skin sections from mice of the indicated genotypes (*n*=4) stained with antibody against CD45 (red) at P0. Nuclei were stained with DAPI (blue). White dashed lines indicate boundary of epidermis (above) and dermis (below). Scale bar, 50 µm. **d,** Gating strategy of $CD45^+$ cells in skin of the indicated genotypes at P0 and P2. Quantification shown in Fig. 3c. **e,** Gating strategy and percentage of the indicated immune cells in skin samples from mice of the indicated genotypes at P2. Data are presented as mean values ± s.e.m (*n*=3 per genotype). **P*≤ 0.05, ****P*≤ 0.001. **f,** Epidermal thickness quantification of skin sections from mice of the indicated genotypes at P0 and P2. Data are presented as mean values ± s.e.m (*n*=3 per genotype). **P*≤ 0.05, ***P*≤ 0.01, NS: not significant. Control mice represent a pool of *Hoip*$^{fl/fl}$;*K14-Cre-* and *Hoip*$^{fl/wt}$;*K14-Cre+* (white circles) or *Hoil-1*$^{fl/fl}$;*K14-Cre-* and *Hoil-1*$^{fl/wt}$;*K14-Cre+* (grey circles). See sequential gating strategy (d, e) in Supplementary Fig. 13.

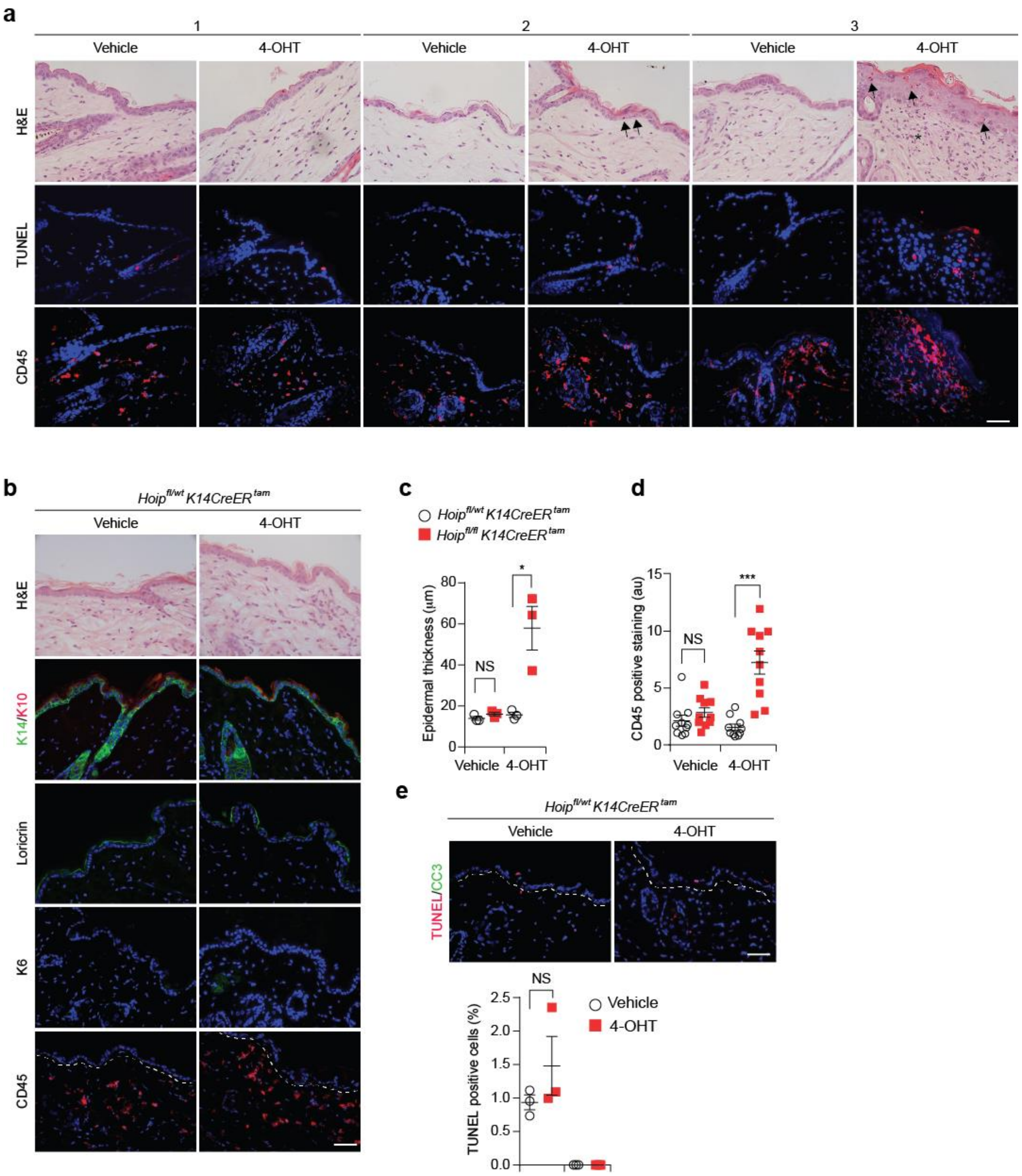

Supplementary Figure 4. Cell death precedes inflammation also when HOIP is deleted in keratinocytes of adult mice. a, b, Representative images of *Hoip*$^{fl/fl}$*K14CreER*^{tam} mice analysed after one, two or three treatments with vehicle or 4-OHT and stained as indicated (a) and of control *Hoip*$^{fl/wt}$*K14CreER*^{tam} mice treated with vehicle or 4-OHT every other day for a total of 4 doses (b) (*n*=3 per genotype). Arrows: pyknotic nuclei, star: immune infiltrates. Nuclei were stained with DAPI (blue). White dashed lines indicate boundary of epidermis (above) and dermis (below) (b). Scale bar, 50 µm. **c,** Epidermal thickness quantification of mice of the indicated genotypes and treated as in (b). Data are presented as mean values ± s.e.m (*n*=3 per genotype). **P*≤ 0.05, NS: not significant. **d**, Quantification of CD45 staining in skin sections from mice of the indicated genotype treated as in (b). au=arbitrary units. Data are presented as mean values ± s.e.m (*n*=10 mice per genotype). ****P*≤ 0.001, NS: not significant. **e,** Representative images of skin sections double stained with TUNEL (red) and cleaved caspase-3 (CC3) antibody (green) from mice of the indicated genotypes (top panels). Nuclei were stained with DAPI (blue). Scale bars, 50 µm. Quantification of TUNEL and CC3 positive cells in skin

sections from *Hoip*$^{fl/wt}$*K14CreER*^{tam} mice treated as indicated (bottom panel) (*n*=3 mice per genotype). Data are presented as mean values ± s.e.m (*n*=3 mice per genotype). NS: not significant.

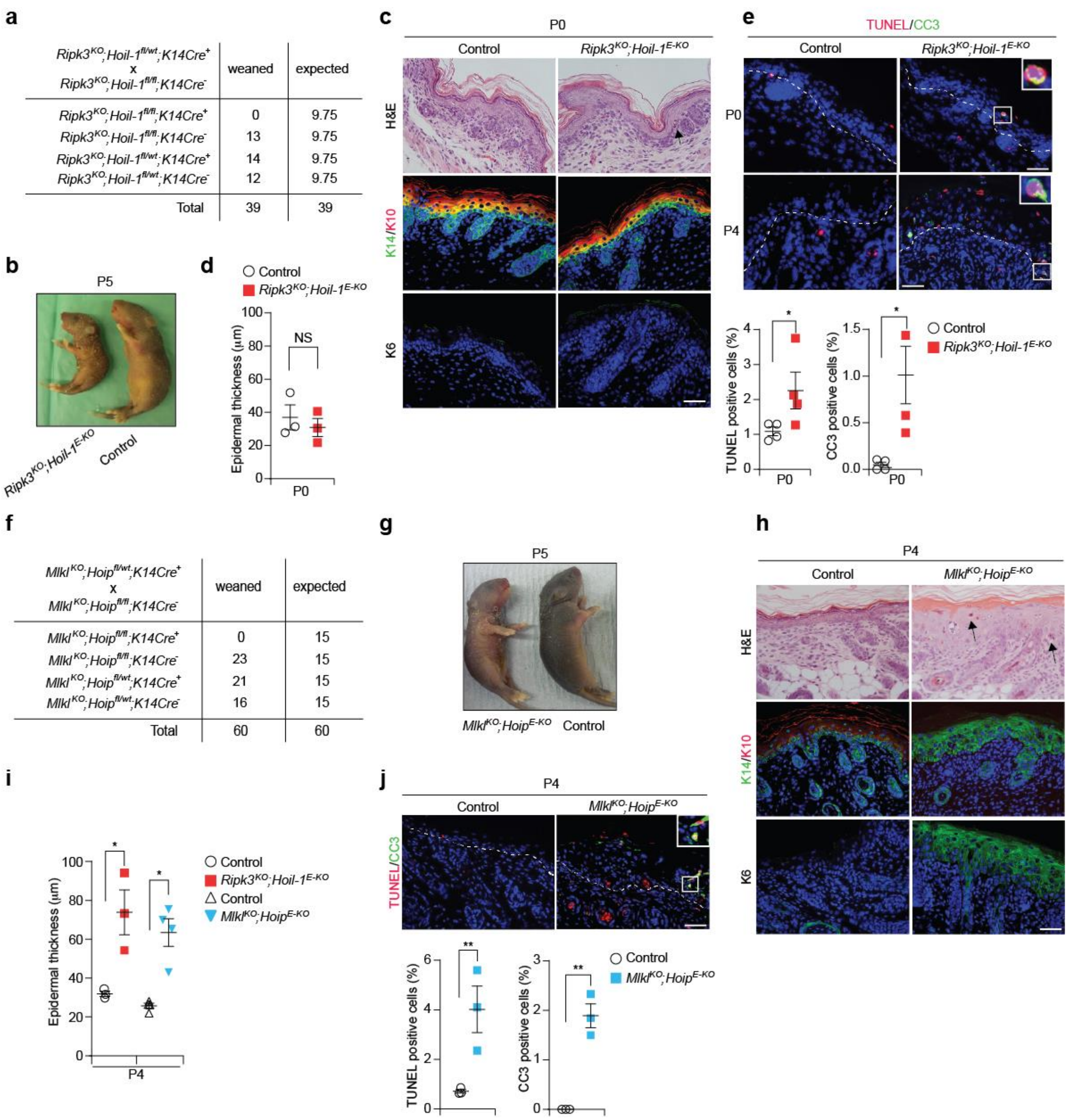

Supplementary Figure 5. Loss of RIPK3/MLKL-mediated necroptosis does not inhibit the severe dermatitis in *Hoip*$^{E\text{-}KO}$ and *Hoil-1*$^{E\text{-}KO}$ mice. a, f, Table depicting genotype statistics of animals obtained after the crossing of mice of the indicated genotypes. Numbers of animals obtained (weaned) and animal numbers expected, according to the Mendelian frequencies, are shown. **b, g,** Representative images of mice of the indicated genotypes at P5. **c, h,** Representative images of skin sections from mice of the indicated genotypes stained as indicated at P0 (c) and at P4 (h) (*n*=4 per genotype). Arrows (H&E) indicate pyknotic nuclei. Nuclei were stained with DAPI (blue). Scale bar, 50 µm. **d, i,** Epidermal thickness quantification of skin sections from mice of the indicated genotypes at P0 (d) and at P4 (i). Data are presented as mean values ± s.e.m (*n*= at least 3 per genotype). **P*≤ 0.05, NS: not significant. **e, j,** Representative images of skin sections double stained with TUNEL (red) and cleaved caspase-3 (CC3) antibody (green) in mice of the indicated genotypes (top panels). Nuclei were stained with DAPI (blue). White dashed lines indicate boundary of epidermis (above) and dermis (below). Scale bars, 50 µm. Quantification of TUNEL and CC3 positive cells in skin sections from mice of the indicated genotypes (bottom panels). Data are presented as mean values ± s.e.m (*n*= at least 3 per genotype). **P*≤ 0.05, ***P*≤ 0.01. Control mice represent a pool of *Ripk3*^{KO};*Hoil-1*$^{fl/fl}$;*K14-Cre-* and *Ripk3*^{KO};*Hoil-1*$^{fl/wt}$;*K14-Cre+* (b-e) and *Mlkl*^{KO};*Hoip*$^{fl/fl}$;*K14-Cre-* and *Mlkl*^{KO};*Hoip*$^{fl/wt}$;*K14-Cre+* mice (g-j).

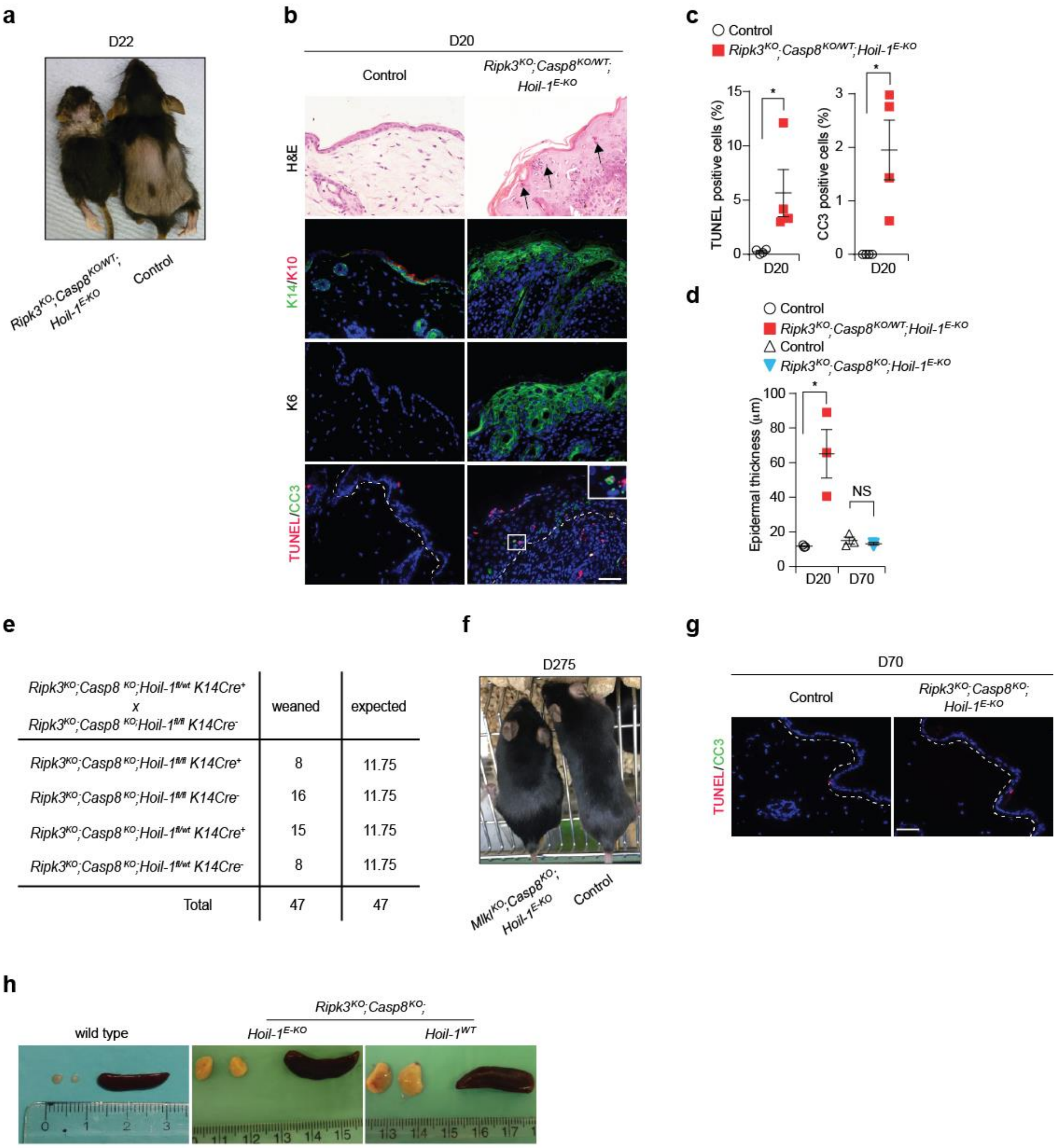

Ripk3^{KO};Casp8 ^{KO};Hoil-1$^{fl/wt}$ K14Cre^{+} x Ripk3^{KO};Casp8 ^{KO};Hoil-1$^{fl/fl}$ K14Cre^{-}	weaned	expected
Ripk3^{KO};Casp8 ^{KO};Hoil-1$^{fl/fl}$ K14Cre^{+}	8	11.75
Ripk3^{KO};Casp8 ^{KO};Hoil-1$^{fl/fl}$ K14Cre^{-}	16	11.75
Ripk3^{KO};Casp8 ^{KO};Hoil-1$^{fl/wt}$ K14Cre^{+}	15	11.75
Ripk3^{KO};Casp8 ^{KO};Hoil-1$^{fl/wt}$ K14Cre^{-}	8	11.75
Total	47	47

Supplementary Figure 6. Contribution of cell death to the severe dermatitis seen in *Hoip$^{E\text{-}KO}$* and *Hoil-1$^{E\text{-}KO}$* mice. a, f, Representative images of mice of the indicated genotypes (*n*=4 (a) and 11 (f) per genotype). **b,** Representative images of skin sections from mice of the indicated genotypes stained as indicated at day 20 (*n*=3 per genotype). Arrows (H&E) indicate pyknotic nuclei. Nuclei were stained with DAPI (blue). White dashed lines indicate boundary of epidermis (above) and dermis (below). Scale bar, 50 µm. **c,** Quantification of TUNEL and CC3 positive cells in skin sections from mice of the indicated genotypes at day 20. Data are presented as mean values ± s.e.m (*n*=4 per genotype). **P*≤ 0.05. **d**, Epidermal thickness quantification of skin sections from mice of the indicated genotypes at the indicated days after birth. Data are presented as mean values ± s.e.m (*n*=3 per genotype). **P*≤ 0.05, NS: not significant. **e**, Table depicting genotype statistics of animals obtained from crossings of mice of the indicated genotypes. Numbers of animals obtained (weaned) and numbers of animals expected, according to the Mendelian frequencies, are shown. **g**, Representative images of skin sections double stained with TUNEL (red) and cleaved caspase-3 (CC3) antibody (green) in mice of the indicated genotypes. Nuclei were stained with DAPI (blue). White dashed lines indicate boundary of epidermis

(above) and dermis (below). Scale bars, 50 µm. **h**, Representative images of axial lymph nodes and spleen from mice of the indicated genotypes at around 7 months. Control mice represent a pool of *Mlkl*^{KO}*;Casp8*^{KO}*;Hoip*$^{fl/fl}$;*K14-Cre-* and *Mlkl*^{KO}*;Casp8*^{KO}*;Hoip*$^{fl/wt}$*;K14-Cre+* (f) or *Ripk3*^{KO}*;Casp8*$^{KO/WT}$*;Hoil-1*$^{fl/fl}$;*K14-Cre-* and *Ripk3*^{KO}*;Casp8*$^{KO/WT}$*;Hoil-1*$^{fl/wt}$*;K14-Cre+* mice or *Ripk3*^{KO}*;Casp8*^{KO}*;Hoil-1*$^{fl/fl}$;*K14-Cre-*, *Ripk3*^{KO}*;Casp8*^{KO}*;Hoil-1*$^{fl/wt}$*;K14-Cre+* (a-d, g).

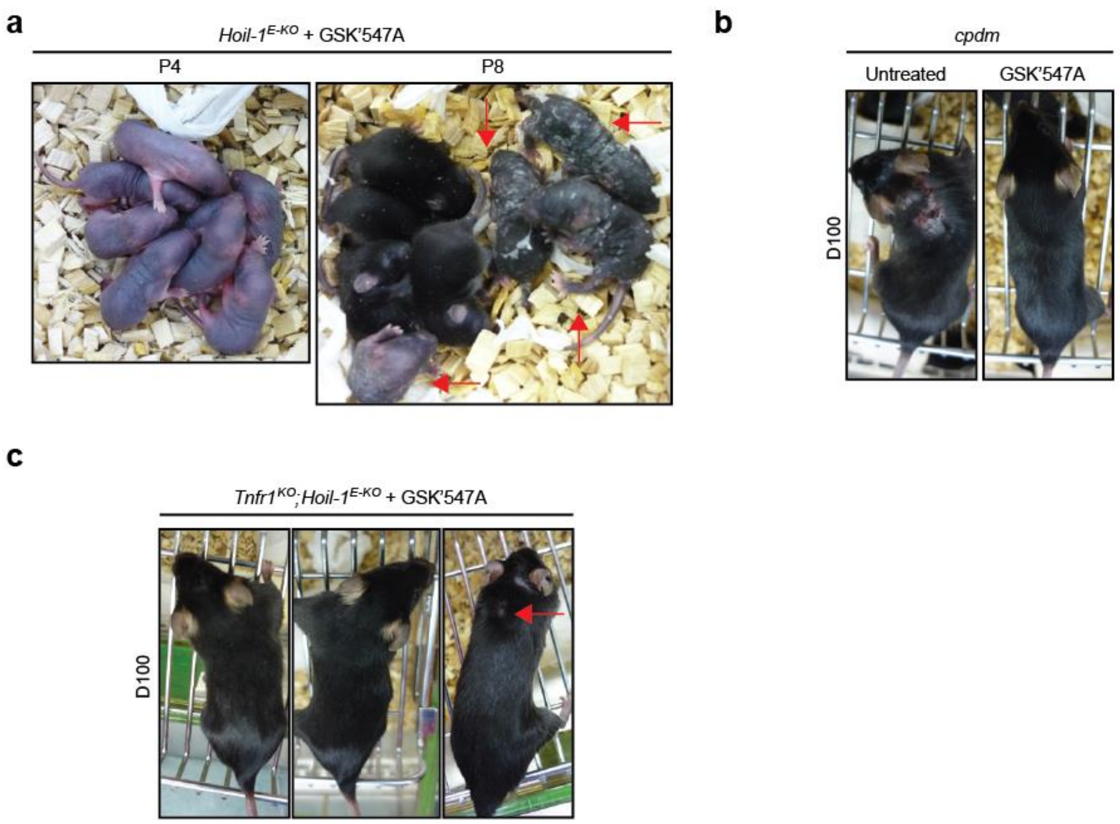

Supplementary Figure 7. Contribution of the RIPK1 kinase activity to the dermatitis induced by absence of LUBAC. a-c, Representative images of mice of the indicated genotypes fed chow containing GSK'547A. Arrows indicate *Hoip*$^{E\text{-}KO}$ mice at P8 (right panel)**;** *Hoip*$^{E\text{-}KO}$ mice were indistinguishable from control littermates at P4 (left panel); control mice represent a pool of *Hoip*$^{fl/fl}$;*K14-Cre-* and *Hoip*$^{fl/wt}$;*K14-Cre+* mice (a). *cpdm* + GSK'547A (*n*=3) (b). Arrow indicates a punctuate crust (c).

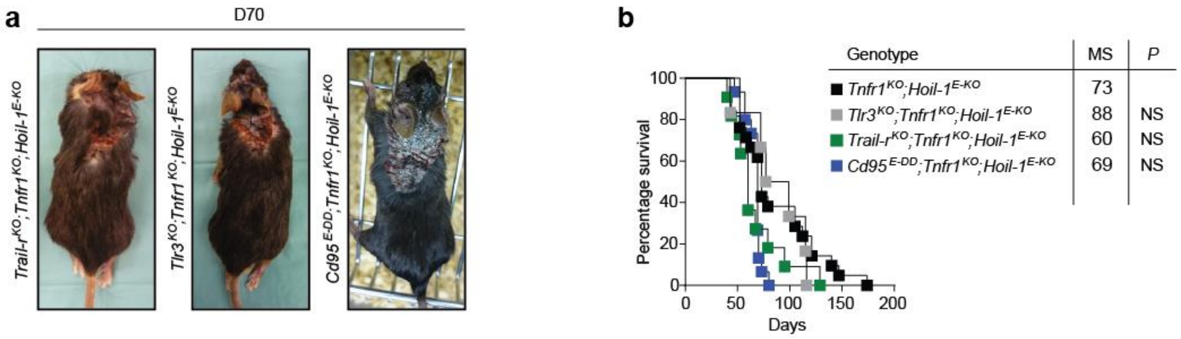

Supplementary Figure 8. Contribution of the death domain (DD) of CD95, TRAIL-R and TLR3 to the dermatitis induced by absence of LUBAC in keratinocytes. a, Representative images of mice of the indicated genotypes. **b,** Kaplan-Meier survival curve, comparison between *Tnfr1*^{KO};*Hoil-1*$^{E\text{-}KO}$ mice and mice of the indicated genotypes were submitted to statistical analysis. *Tnfr1*^{KO};*Hoil-1*$^{E\text{-}KO}$ (*n*=21), *Trail-r*^{KO};*Tnfr1*^{KO};*Hoil-1*$^{E\text{-}KO}$ (*n*=11), *Tlr3*^{KO};*Tnfr1*^{KO};*Hoil-1*$^{E\text{-}KO}$ (*n*=6) and *Cd95*$^{E\text{-}DD}$;*Tnfr1*^{KO};*Hoil-1*$^{E\text{-}KO}$ (*n*=15). MS: median survival, NS: not significant.

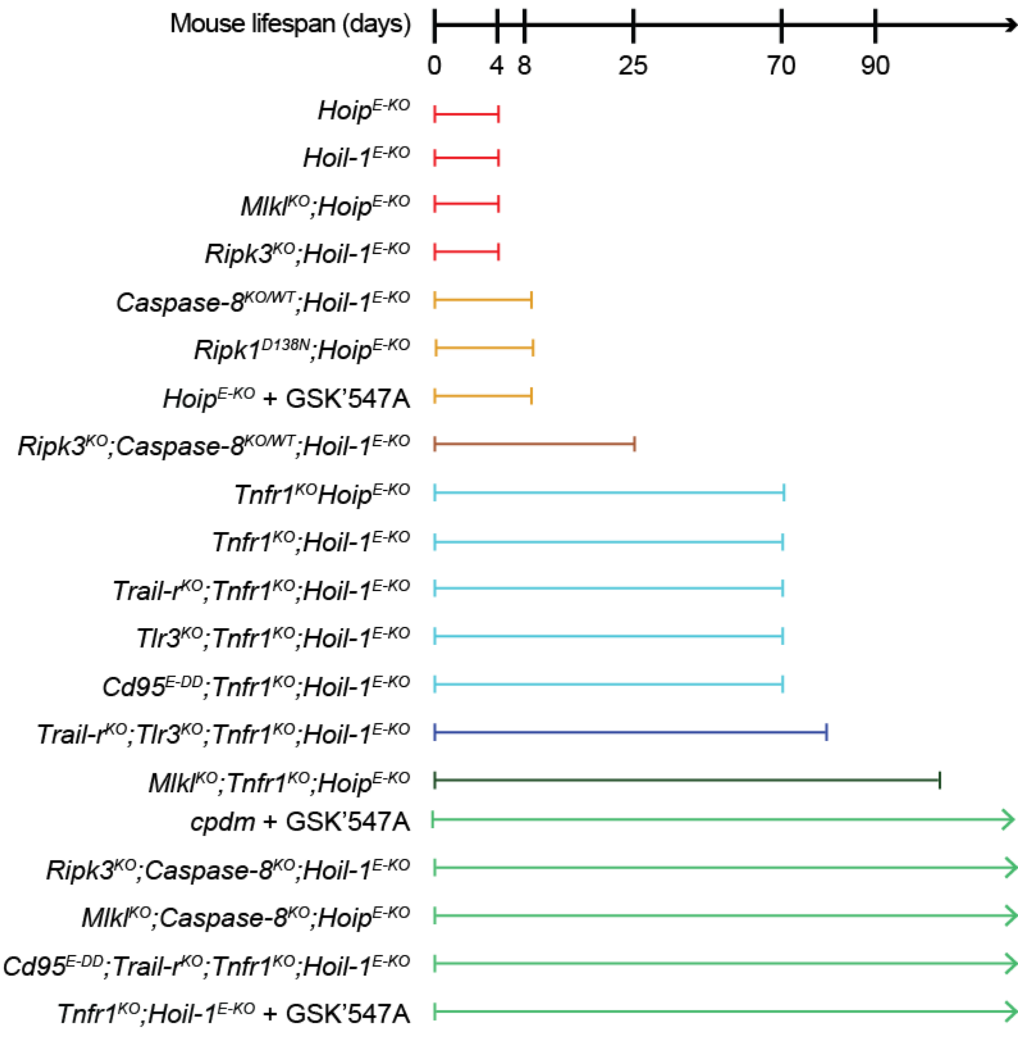

Supplementary Figure 9. Summary of mice lifespan. Median survival of mice with indicated genotypes.

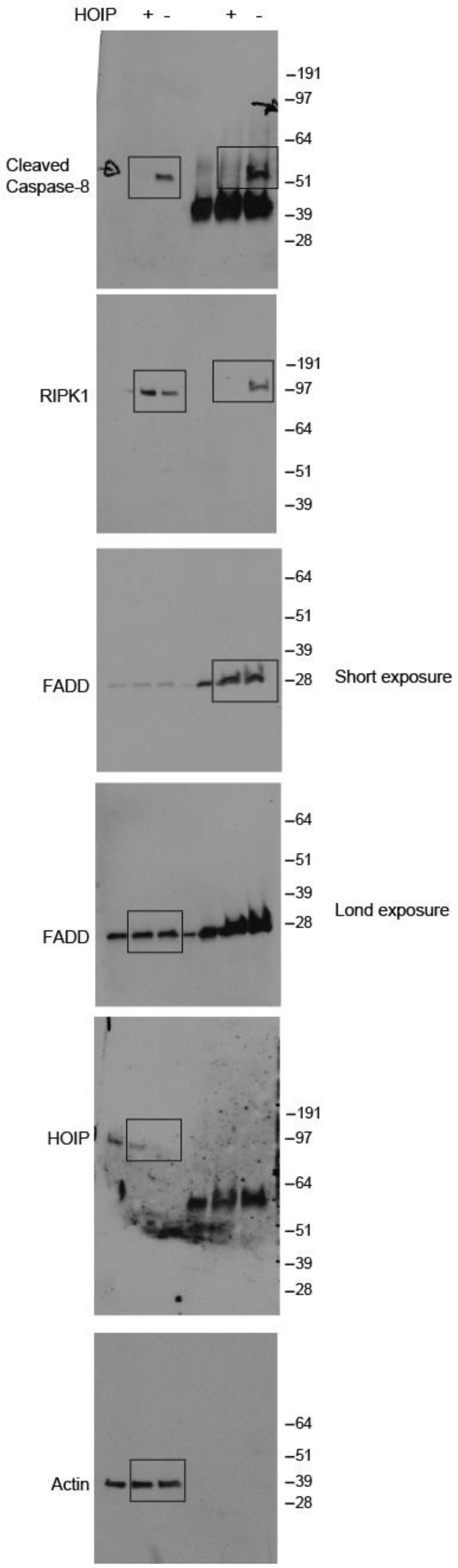

Supplementary Figure 10: Uncropped blots from Figure 4a

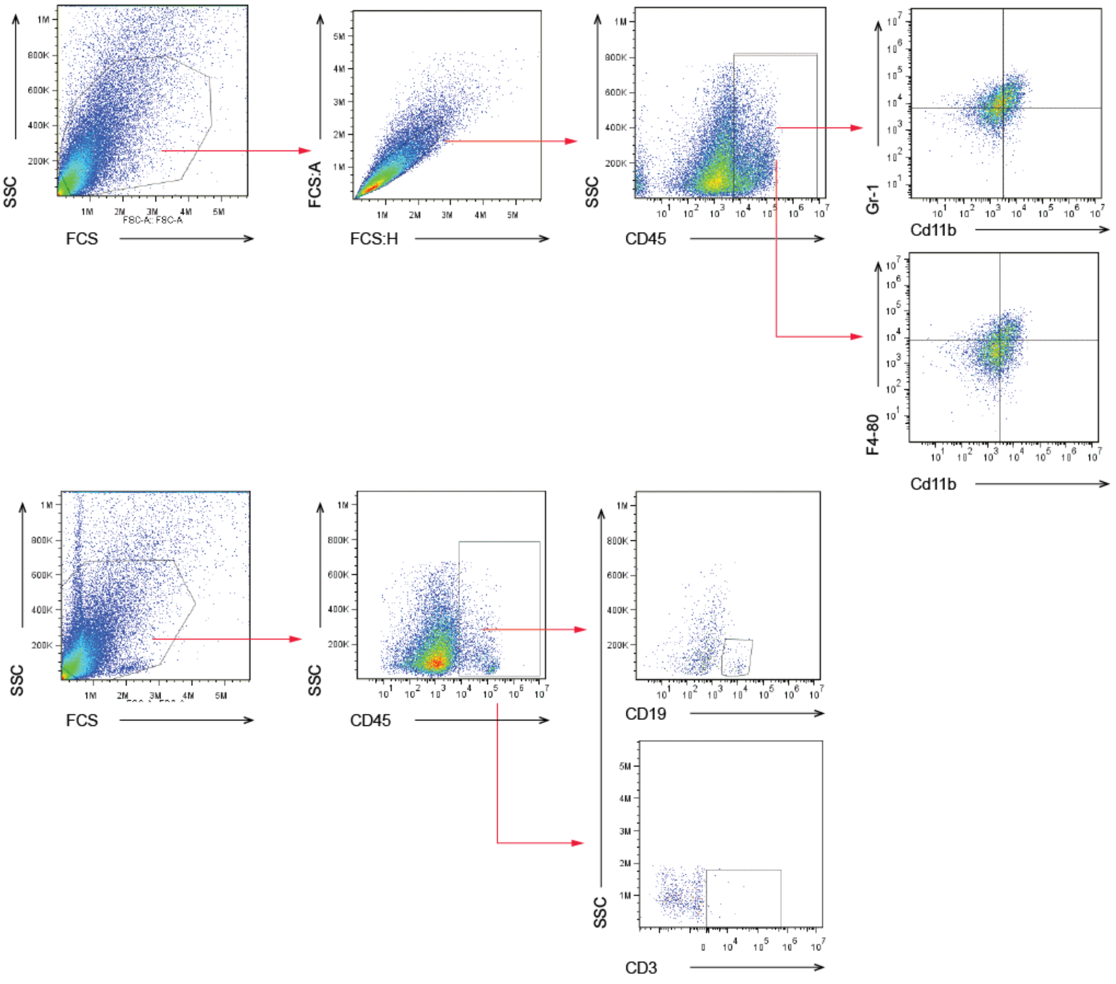

Supplementary Figure 11: Gating strategy corresponding to Figure 1 d and Supplementary Figure 1f, g.

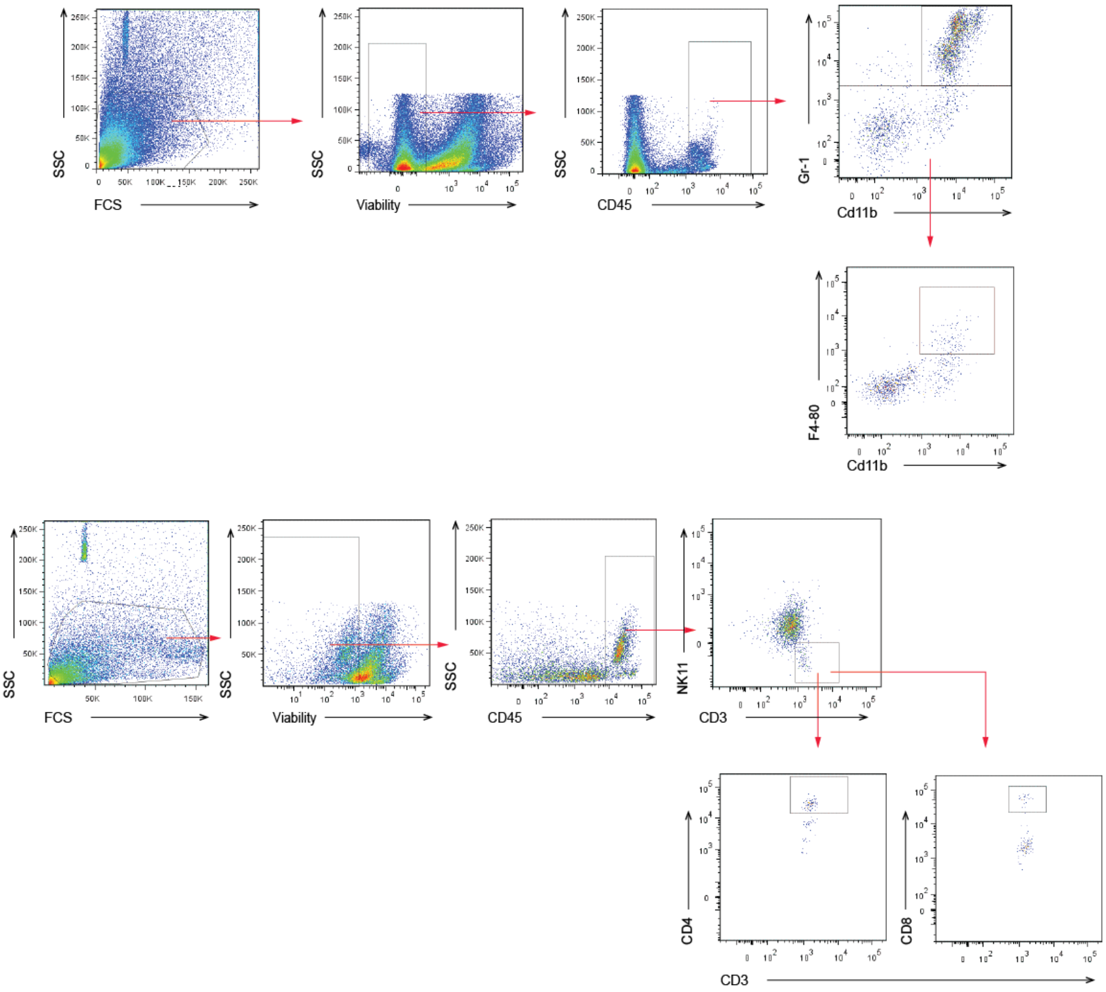

Supplementary Figure 12: Gating strategies corresponding to Figure 2d and Supplementary Figure 2b-d.

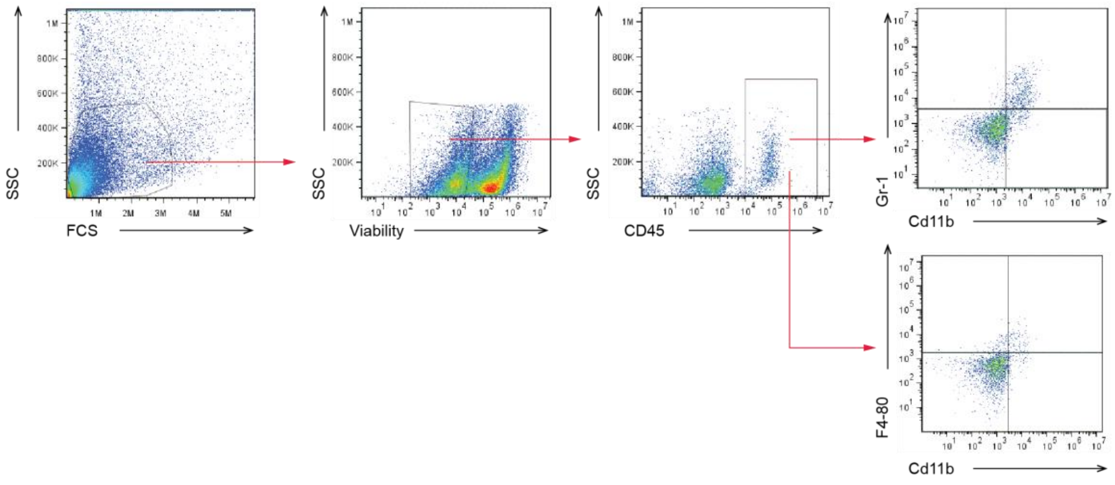

Supplementary Figure 13: Gating strategies corresponding to Figure 3c and Supplementary Figure 3e.
